# The Structure of the Relationship between Physical Activity and Psychosocial Functioning of Women and Men during the COVID-19 Epidemic in Poland

**DOI:** 10.3390/ijerph191911860

**Published:** 2022-09-20

**Authors:** Anna Mazur, Elżbieta Bartoń

**Affiliations:** 1 Laboratory of Psychoprophylaxis and Psychological Support, Faculty of Human Science, University of Economics and Innovation, 20-209 Lublin, Poland; 2 Department of Neurology, Neurological and Psychiatric Nursing, Chair of Conservative Nursing, Medical University, 20-059 Lublin, Poland

**Keywords:** COVID-19 epidemic, gender, physical activity, psychosocial functioning

## Abstract

Since the coronavirus disease (COVID-19) pandemic is a serious crisis in many countries around the world, it is important to conduct empirical research aimed at identifying risks and factors protecting the functioning of people affected by it. For this reason, the goals of the present research were to determine the level of physical activity and the severity of symptoms characteristic of mental disorders, cognitive disorders and the quality of social functioning, as well as the structure of the relationship between physical activity and psychosocial functioning of 226 women and 226 men during the COVID-19 epidemic in Eastern Poland. The research was conducted using the *IPAQ-SF Questionnaire*, *GHQ-28 Questionnaires*, *TUS Test-6/9 version*, the original *SFS Scale* and a self-developed sociodemographic survey. The collected data indicate that women as compared to men show lower levels of weekly physical activity, walking, moderate activity, vigorous activity and quality of functioning in family relationships, but higher severity of mental health disorders, somatic symptoms, functional disorders, depressive symptoms, cognitive disorders, perceptual work disorders, attention deficits and higher quality of functioning in work relationships. On the other hand, the structural model indicates that physical activity, interacting with mental health disorders and cognitive disorders, is positively associated with the social functioning of the respondents, and gender is the moderator of the occurring dependencies. This suggests that physical activity adapted to the condition of health may be an important component of gender-individualized psychopreventive interventions.

## 1. Introduction

The leading health and social problem in many countries around the world is the coronavirus (COVID-19) pandemic. Up to 6 August 2022, a total of 588,081,229 cases of disease were recorded in various regions of the world, including 6,434,821 deaths and 559,178,151 recoveries. In Poland, the reach of the epidemic is 6,093,571 people, of which 116,660 patients have died and 5,335,862 have already been cured [1].

Empirical evidence from different countries affected by the pandemic suggests that symptoms of anxiety and depression (16.0–28.0%) and sleep disorders (38.9%), which are associated with impaired cognitive dynamics and cognitive deficits, including attention and perceptiveness [2,3], are relatively frequent psychological reactions to the current crisis situation, with higher rates of symptoms of mental health disorders found in women than in men [4,5,6]. On the other hand, both the occurrence of the above-mentioned symptoms and the consequences of life limitations during the pandemic may disrupt the social life of many people, and thus lower the quality of their relationships with family members and friends and professional interactions [6,7]. 

When attempting to understand the nature of the aforementioned crisis, it is important to refer to the leading assumptions of the biopsychosocial model dominating in modern health sciences, which can explain intrapsychic reactions of people to the pandemic as a result of the interaction of a network of equivalent biological, psychological and social factors shaping health [8,9,10]. 

Taking into account the biological factors included in the considered model, it should be emphasized that empirical evidence from various countries affected by the pandemic indicates that gender moderates the resistance to stressors and has a significant influence on psychological variables important in coronavirus (SARS-CoV-2) transmission, specifically mental health and cognitive processes [8,9,10,11,12]. On the basis of a meta-analysis, it was proven that women exhibit a higher intensity of symptoms characteristic of depressive syndrome and anxiety disorders than men associated with the presence of attention and perceptiveness deficits, which may indicate that it is more difficult for them to cope with the current situation [13,14,15,16,17]. 

It was also observed that the mental condition and the state of cognitive processes during the current crisis may be of significant importance for social functioning, including family, interpersonal and professional interactions [18]. It is postulated that the quality of these relationships is directly related to the received social support, acting as a factor protecting against the negative consequences of experienced stress [19]. 

It has been observed that during the transmission of SARS-CoV-2, women report a greater sense of loneliness than men [15,17]. Moreover, regardless of gender, in many countries, including Poland, Spain, Australia, the United States, Sweden and China, due to social isolation, quarantine, working from home and other pandemic restrictions, the number of social interactions decreased [15,17,20,21,22]. This at the same time reduces the likelihood of receiving support, and thus increases the risk of developing unwanted psychological symptoms, including depression, anxiety disorders and accompanying problems in cognitive functioning [23].

Bearing in mind that, according to the adopted approach, a human is a living system, constituting an indivisible whole, composed of biological, psychological and social dimensions, which, under the influence of incoming information, or, in other words, the requirements of the environment, remain in a dynamic balance, it is extremely important to pay attention to factors supporting its remedial efficiency, and their consequences on the general health condition [9,10]. 

One of the key forms of preventive action recommended by the World Health Organization (WHO) is regular physical activity, defined as any type of body movement initiated by working muscles that causes energy expenditure. It includes walking, moderate and vigorous activity, which, at the same time, strongly distinguishes it from other forms of leisure activities, including time spent on passive leisure such as sitting and lying down [24,25].

The available literature shows that physical activity understood this way reduces the risk of developing undesirable mental symptoms as well as disorders in cognitive functioning, because by increasing the metabolism of kynurenine and the expression of kynurenine aminotransferase in skeletal muscles, it minimizes the consequences of environmental stress, and thus prevents the appearance or reduces the severity of symptoms characteristic to neuropsychiatric disorders [26].

It has been proven that systematic physical exertion delays neurodegenerative processes and regulates monoamine metabolism, as well as neuroimmune functioning [27]. The physiological changes taking place under the influence of training support the proper functioning of the hypothalamic–pituitary–adrenal axis (HPA), the excessive activation of which, caused by increased and sustained release of cortisol, is associated with the risk of developing many disorders [28,29,30]. In addition, physical activity by stimulating the expression of the brain nerve growth factor (BDNF), as well as supporting the proper functioning of the Trk-B receptor and BDNF-Trk-B signaling, determines the neurophysiological processes taking place, which in turn has a beneficial effect on affective, cognitive and social functioning [31,32].

The vast majority of studies indicate that men have higher levels of physical activity than women, which may be due to different uses of the time available in the course of a day [33,34,35,36]. A nationwide study reported that, on average, men devote one hour and thirty-three minutes per day to sports, while women, on average, perform physical exercises for one hour and nineteen minutes per day. Moreover, men’s workouts tend to be characterized by higher intensity than those performed by women. As a result, men’s higher physical activity, to a greater extent than women’s, may be a factor in protecting their mental fitness [35,36].

It is worth mentioning, however, that the results of studies conducted in different regions of Poland are not consistent. For example, in the population of residents of northeastern Poland, it has been shown that, although moderate physical activity is undertaken more often by men (53.9%) than by women (34.7%), and that men, compared to the opposite sex, are almost twice as likely to ride a bicycle (31.5% vs. 13.1%), but at the same time it has been observed that, regardless of gender, the physical activity of residents of this region is insufficient and differs significantly from levels recorded in other parts of the country, as well as in other European countries [33].

Results of a cross-sectional study conducted in a random sample of 6000 people between the ages of 40 and 64 in Japan are also of interest. They came to slightly different conclusions than the empirical works cited above. They observed that women, on average, spent 12.6% less time on sedentary lifestyles and 23.4% more time on low-intensity physical activity than men, but found no significant differences for moderate and vigorous physical activity [37].

This suggests that the conclusions of the empirical works published to date are inconclusive. The reported results vary depending on the region of Poland, as well as the part of world in which they’re conducted [33,34,35,36,37], which at the same time suggests that they appear to be worth supplementing. The results of our own research will therefore allow us to determine whether, and possibly to what extent, the observations of other researchers reflect the situation of the people of Eastern Poland. Considering that there are no studies aimed at determining the level of physical activity, mental health, cognitive processes and quality of social functioning, as well as the importance of interactions between these variables in the population of women and men exposed to stress resulting from the COVID-19 epidemic in Eastern Poland, the data obtained can provide both valuable scientific observation and be used to develop psychoprophylaxis interventions.

Hence, the aim of this own research was to determine the level of physical activity, mental health, cognitive processes and quality of social functioning, as well as the structure of the relationship between physical activity and psychosocial functioning of women and men during the COVID-19 epidemic in Eastern Poland.

Two research questions were posed accordingly: (1) what is the level of physical activity, mental health disorders, cognitive disorders and social functioning, as well as (2) what is the significance of the interactions between physical activity, mental health and cognitive disorders for the social functioning of men and women during the COVID-19 pandemic in Poland?

In response, two hypotheses were formulated, assuming that during the COVID-19 epidemic in Poland, depending on gender, the following varies: (1) the level of physical activity, the intensity of symptoms characteristic of mental health disorders, cognitive disorders and the quality of social functioning as well as (2) the importance of the interaction between physical activity, mental health and cognitive disorders for social functioning.

## 2. Material and Methods

### 2.1. Design

While conceptualizing the author’s own research, the biopsychosocial model of health was used [8,9,10]. Based on the above theoretical frameworks, in the quasi-experimental model, a cross-sectional research plan was designed.

In line with the adopted theoretical approach, in this research project, variables of biological (gender), psychological (physical activity, mental health disorders, cognitive disorders) and social (social functioning) factors were distinguished, which were assumed to interact with each other.

Whereas the prognostic factors of mental health and cognitive disorders, as well as the social functioning received, may vary depending on country and even region [12,38,39,40], the analysis focused on the consequences of the epidemic in the population of Eastern Poland, which means that the surveyed sample included only the inhabitants of these areas. Additionally, the concept takes into account the moderating role of gender, as it is an important biological determinant of susceptibility to physical activity, mental and cognitive disorders and social functioning [11,12,14,15,41].

It was assumed that the mentioned parameters are latent variables, which is characteristic of fully interactive approaches and at the same time allows for a detailed analysis of the mutual and multifaceted interaction of individual factors creating the structure of physical activity, as well as shaping the state of mental health, cognitive processes and social functioning [8,9,10,42].

Given that the biopsychosocial model of health [9], as well as numerous empirical premises, suggests a significant role of sociodemographic factors for the variables analyzed in the study, during the conceptualization of the research, many controlled variables, which may potentially influence the results, have been taken into account [13,43,44]. They include: gender [14,15], age [43], place of residence [38], level of education [45], marital status, number of children [15,46], professional activity [45] and health condition of the respondents [7,47,48].

In order to minimize the influence of gender on the obtained research results, the analyzed groups were of the same size. In the case of a health condition, a procedure based on establishing its constant level was applied. Age, marital status, place of residence and professional activity were checked by designating their subranges, and, as in the case of the other above-mentioned variables, the homogeneity of the studied groups was confirmed [42].

In the first stage of the analysis, the level of physical activity, mental health disorders, cognitive disorders and social functioning of women and men during the epidemic were assessed. In the next stage, the focus was on verified gender-related occurrence and significance of the interactions and relationships between the identified variables based on the modeling of structural equations in the confirmatory version.

The research was carried out in the population of adults of Eastern Poland in the period from June, 2020 to December, 2021. In total, 579 people declared their willingness to participate, and 452 women and men met the inclusion criteria (78.7%). Before and during the study, the participants showed no symptoms suggesting a risk of SARS-CoV-2 infection and were not quarantined.

The research was conducted in accordance with the guidelines of the Helsinki Declaration and received approval from the Bioethics Committee of the Medical University of Lublin (No. KE-0254/100/2020, 28 May 2020). The persons obtained all necessary information and explanations about the project. In addition, they were informed about the confidentiality of the data provided and about the possibility of receiving feedback based on individual results. In case of doubts, they had the opportunity to obtain additional explanations.

Due to the risk of SARS-CoV-2 infection and restrictions introduced in the country, each person was contacted online. The invitation to participate in the research along with contact details (telephone number, first and last names of persons to contact) was posted on the Internet on the website of NeuroCentrum [49]. It was available from 1 June 2020 to the end of November 2021. The invitation to participate in the study was extended to adult residents of Eastern Poland according to the adopted inclusion and exclusion criteria.

These inclusion criteria were: free and informed written consent to participate in the research, age (adults), origin (inhabitants of Eastern Poland), marital status (married or partnership), employment based on an employment contract or contract of mandate, no diagnosis of COVID-19 and a lack of SARS-CoV-2 symptoms, as well as good general psychophysical health. The exclusion criterion was: failure to meet at least one of the above-mentioned inclusion criteria.

Participants were recruited by phone. Those who declared their willingness to participate in the research, and at the same time met the inclusion criteria, provided the recruiter with a telephone number and an address. They were mailed an informed consent form necessary to participate in the study, along with written information about the research and a return envelope enabling the documents to be sent back. After receiving them, the individuals were contacted by phone so a convenient date to carry out the study could be set.

The questionnaire session was carried out during individual video conferences. After acquainting respondents with the rules of completing the questionnaire items, a sheet was presented to them with individual questions, and the range of possible answers were read, and answers were marked in the appropriate place on the test. On average, the research period lasted for 20 min.

### 2.2. Participants

The sample consisted of two groups of inhabitants of Eastern Poland (*n* = 452). The first was made up of women (*n* = 226), and the second was made up of men (*n* = 226). The participants were 18–80 years old. Detailed sociodemographic characteristics of the respondents by gender are presented in Table 1.

The analyzed groups were homogeneous in terms of age, number of children, place of residence, education, marital status and professional activity.

### 2.3. Physical Activity Measurements

The Short Form *Physical Activity Questionnaire* (*IPAQ-SF*) was used to measure weekly physical activity. It contains seven questions about the time spent on activities that require vigorous and moderate physical effort, as well as time spent on walks and time spent in the form of passive rest (sitting, lying down). Weekly exercise level can be estimated in MET units (minute/week). It is the product of the activity coefficient for a specific activity, the number of active days and its duration in minutes. On this basis, the level of physical activity of the examined person is determined, which may be: (a) high—from 3 to 7 days of intense physical exertion or at least 1500 MET; (b) sufficient—3 to 7 days of vigorous physical activity for at least 20 min a day or 5 to 7 days of moderate exercise/walking for at least 30 min a day or 5 to 7 days of any physical activity that in total exceeds 600 MET; (c) insufficient—lack of physical activity or failure to meet the conditions of the two above-mentioned levels [25,50].

### 2.4. Mental Health Measurements

The *General Health Questionnaire* (*GHQ-28*) was used to measure mental health. The tool is used to assess mental health, which is divided into four dimensions: somatic symptoms, anxiety and insomnia, social dysfunction and symptoms of depression. The *GHQ-28* enables the identification of people whose mental condition has been subject to a temporary or long-term breakdown as a result of experienced difficulties or as a result of mental illness, and those who are at a significant risk of mental health disorders. The cut-off point is a score of 12 or more. The questionnaire has high internal consistency and validity rates. The values of Cronbach’s *alpha* coefficients, calculated in the studied population of women, ranged from 0.76 to 0.82, and in the population of men ranged from 0.76 to 0.83 [51].

### 2.5. Cognitive Functioning Measurements

The *Attention and Perceptiveness Test* (*TUS*) version 6/9 was used to measure cognitive functioning in terms of attention and perceptiveness. The tool is used to assess cognitive disorders in three dimensions: perceptual work, perception deficits and attention deficits. The *TUS* enables the identification of people whose cognitive functioning condition has been subject to a temporary or long-term breakdown as a result of experienced difficulties or as a result of illness, and those who are at a significant risk of cognitive disorders. The questionnaire has high internal consistency and validity rates. The values of the absolute stability coefficients (3-week interval between tests), calculated in the studied population of women, ranged from 0.77 to 0.81, and in the population of men ranged from 0.78 to 0.83. The results were converted to sten scores [52].

### 2.6. Social Functioning Measurements

To measure social functioning during the COVID-19 epidemic, the *Social Functioning Scale* (*SFS*) was used. This scale was constructed for this study. The tool was developed on the basis of Falvo’s [18] concept of social functioning. The *SFS* measures the quality of social functioning during the COVID-19 epidemic in three spheres, which include family and interpersonal and professional relationships. The test contains 21 questions that respondents refer to on a six-point Likert scale. The answers range from *definitely no* to *definitely yes*. The test validation and normalization procedure were carried out on a sample of 700 adults. The content validity coefficients of the *CVR* ranged from 0.96 to 1.00. The theoretical validity was verified by exploratory factor analysis with simple Oblimin rotation and Kaiser normalization and confirmatory factor analysis. Criterion validity was estimated on the basis of the analysis of the correlation matrix with dimensions theoretically related to social functioning: self-esteem, life satisfaction, social support and the results of other tests measuring social functioning. Cronbach’s *alpha* coefficients in the group of studied women ranged from 0.81 to 0.88, and in the population of men, from 0.83 to 0.90. The results were converted to sten scores.

### 2.7. Measurements of Controlled Variables

For the measurement of controlled variables, a questionnaire created for the needs of the conducted research was used. It consisted of a series of questions about sociodemographic data (gender, age, place of residence, education status, marital status, number of children, professional activity) and the health condition of the respondents.

### 2.8. Statistical Analysis

Statistical calculations were performed on anonymized data using IBM SPSS 27 with AMOS software.

The description of the researched sample was based on the calculation of the percentage distribution of the qualitative data frequency, the mean values, standard deviation and minimum and maximum values of quantitative parameters.

The significance level of intergroup differences in terms of qualitative data was verified using the *c*^2^ test. The reliability of the tests was estimated based on the Cronbach *alpha* measures.

The shape of the variable distributions was estimated on the basis of the Kolmogorov–Smirnov test. Levels of activity of the research subjects were compared using a *c*^2^ test, while the effect size was determined based on Cramer’s *V* value. The verification of intergender differences was carried out using Student’s *t*-test for independent data, the statistically significant results of which were supplemented by estimating the size of the effects using Cohen’s *d* coefficient.

The characteristics of the physical activity of the respondents, their mental health, cognitive functioning and social functioning were determined on the basis of the mean value, standard deviation and minimum and maximum values.

The estimation of measurement models and the interactive structural model was performed using SEM structural equation modeling in the confirmatory version, in which the *Maximum Likelihood* method was used. The use of an advanced method of statistical analysis made it possible to verify the occurrence and significance of mutual and multifaceted interaction of individual factors forming the structure of physical activity for women and men, shaping the state of mental health and cognitive disorders, as well as social functioning. In the presented models (measurement and structural), the values placed next to the arrows indicate the estimates of standardized coefficients for a given path, while the values above the observable variables represent the constants in the equation. The coefficients for random factors (variables e) are initially arbitrarily set at the level of 1 as non-standardized, and after standardization, their values are equal to 0 and are not presented.

In the work, the boundary point of committing a type I error is 0.05.

## 3. Results

### 3.1. Intergroup Comparisons

In the first step of the conducted analysis, levels of weekly physical activity of the subjects were compared. Results are shown in Table 2.

Nearly three-quarters of the women surveyed showed a sufficient level of weekly physical activity. A high level of activity characterized 15.0% of female respondents, and a low level of activity characterized more than one-tenth of female respondents.

In the group of men, more than three-quarters of the study participants were characterized by a high level of weekly physical activity. The activity of nearly one-fifth of the respondents was sufficient, while the lowest percentage of men showed insufficient weekly physical activity.

The analysis resulted in a strong statistically significant gender effect for weekly physical activity.

Women, compared to men, less often revealed a high level of physical activity, while more often, their weekly activity was classified as sufficient and insufficient.

In the second step of the analysis, the level of physical activity, mental health, cognitive processes and the quality of social functioning of the surveyed women and men were assessed. The results of the comparative analysis are presented in Table 3.

The weekly physical activity of the surveyed women was classified as sufficient. However, in the male population it reached a high level. Regardless of gender, the respondents spent the most time on walks, then on intense physical activity, and the least on training with a moderate level of vigorous activity and passive rest (sitting, lying down).

The general mental health of the study participants was within the upper limit of the average results, which also suggests that in the analyzed population of women, in the event of the occurrence of adverse factors generating severe and chronic stress, there may be a risk of developing symptoms characteristic of mental health disorders. In women, somatic symptoms were most severely intensified, followed by anxiety and insomnia, symptoms of depression and symptoms typical of disorders in everyday functioning. In the group of men, the overall mental health condition was moderate. The respondents were dominated by a sense of anxiety and insomnia, while functional disorders, somatic symptoms and symptoms of depression were less severe.

The functioning of the cognitive processes of the studied populations was classified as moderate. In the female population, the most pronounced difficulties related to perceptual work, followed by perception deficits and attention deficits. The highest intensity of perception deficits was observed in men, while the problems with perceptual work and attention deficits were at a slightly lower level.

Social functioning of the analyzed groups was in the area of moderate results. Women did best in family relationships and among acquaintances and friends, and the worst in professional interactions. Men showed the highest level of coping skills in interpersonal relationships, then in family relationships, and the poorest quality of interactions in professional relationships.

As a result of the comparative analysis, a strong gender effect was noted for moderate activity; moderate gender effects for weekly activity, somatic symptoms, cognitive functioning, perceptual work, attention deficits and professional relationships; and weak gender effects for walking, vigorous activity, mental disorders, functional disorders, depressive symptoms and family relationships.

Compared to men, women showed a lower level of weekly physical activity, walking, moderate physical activity and vigorous physical activity, as well as a lower quality of family relationships. Moreover, more severe mental disorders, somatic symptoms, functional disorders, depressive symptoms, cognitive disorders, perceptual work disorders, attention deficits and a higher quality of relationships in the area of professional life were more often noted in the female population than in men. On the other hand, sitting and lying down, anxiety and insomnia, perception deficits, social relationship and interpersonal relationships were at a comparable level in the analyzed groups.

### 3.2. Estimation of Measurement Models

Prior to estimation of the complete interactive structural model presenting the relationships between physical activity, mental health, cognitive disorders and social functioning, its four measurement parts were estimated. Model specifications were carried out in the group of women and men.

**Figure 1:**
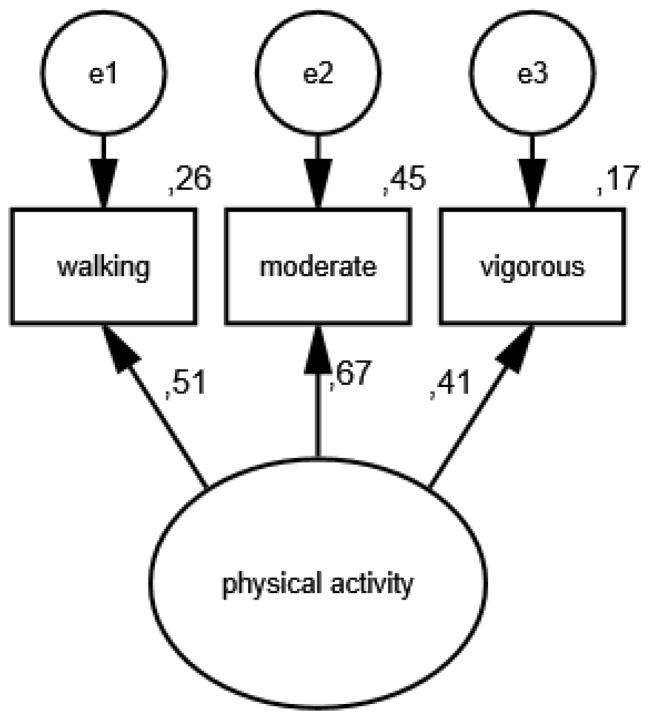
Standardized effects of the model of physical activity measurement in the group of women.

**Figure 2:**
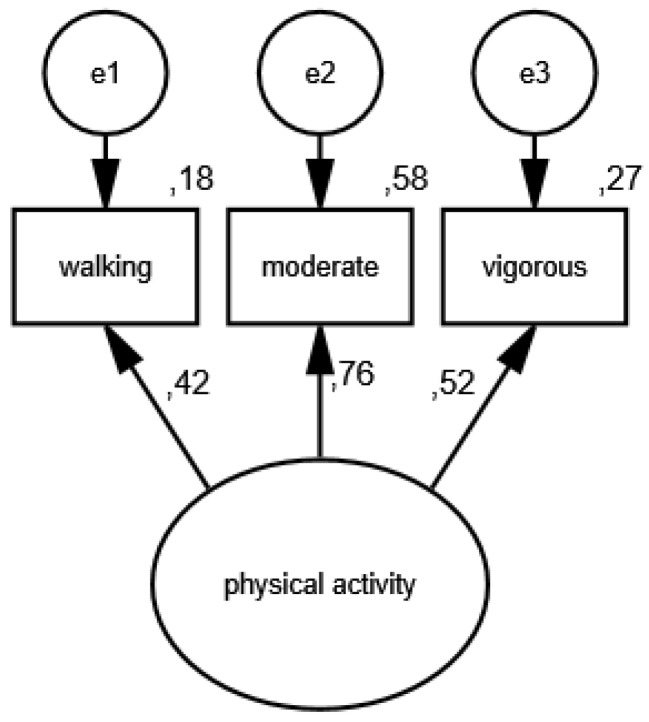
Standardized effects of the model of physical activity measurement in the group of men.

In the first measurement model, physical activity is a latent variable, while its partial indicators are walking, moderate activity and vigorous activity. Figure 1 shows the results of verification of the constructed model of physical activity measurement in the group of women, and Figure 2 illustrates the measurement model of physical activity in the group of men.

As a result of the estimation, the following measures were obtained: CMIN/DF = 1.75; GFI = 0.99; AGFI = 0.99; NFI = 0.99; RFI = 0.95; IFI = 0.99; TLI = 0.99; CFI = 0.99; RMSEA = 0.001(0.001; 0.05); PCLOSE = 0.92; *N* HOELTER(1269; 1561), which confirm that the model reflects the nature of the relationships between the analyzed variablesvery well. The standardized values of factor loadings and the percentages of variances explained in each of the analyzed groups show that the constructed model of physical activity measurement is acceptable.

**Figure 3:**
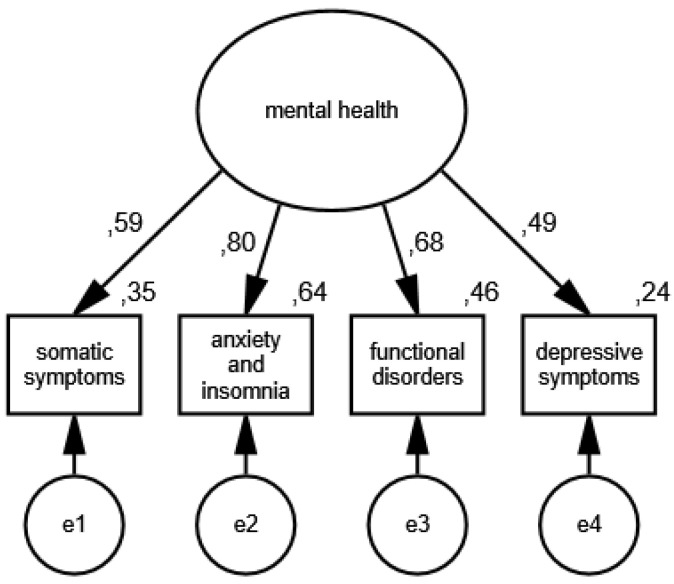
Standardized effects of the mental health disorder measurement model in the group of women.

**Figure 4:**
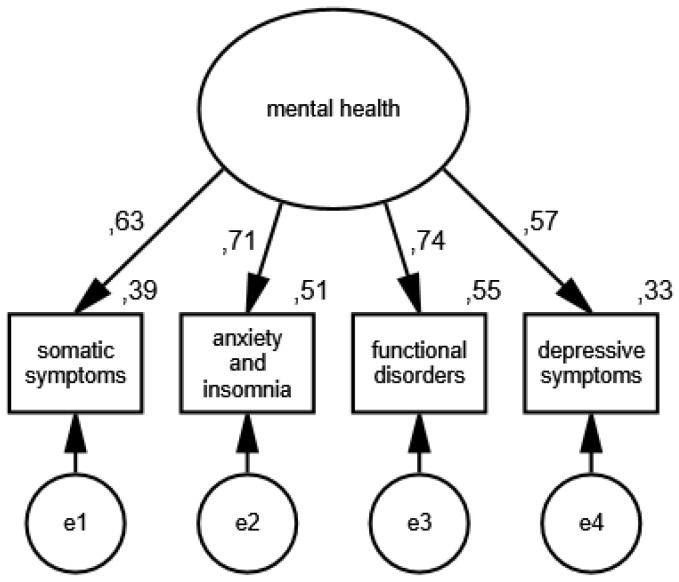
Standardized effects of the mental health disorder measurement model in the group of men.

In the second measurement model, the presence of mental health disorders is an unobservable variable, and its partial indicators are somatic symptoms, anxiety and insomnia, functional disorders and the symptoms of depression. The results of the verification of the constructed model for measuring mental health disorders in the group of women are shown in Figure 3 and Figure 4 illustrates the measurement model of mental health disorders in the group of men.

The following measures were obtained during the verification of the model: CMIN/DF = 1.75; GFI = 0.99; AGFI = 0.99; NFI = 0.99; RFI = 0.95; IFI = 0.99; TLI = 0.99; CFI = 0.99; RMSEA = 0.001(0.001; 0.05); PCLOSE = 0.92; *N* HOELTER(1116; 1442), which confirm that it is correctly fitted to the data, and the standardized values of factor loadings and the percentages of variances explained in the group of women and men allow for recognizing the constructed model of mental health disorders measurement as acceptable.

**Figure 5:**
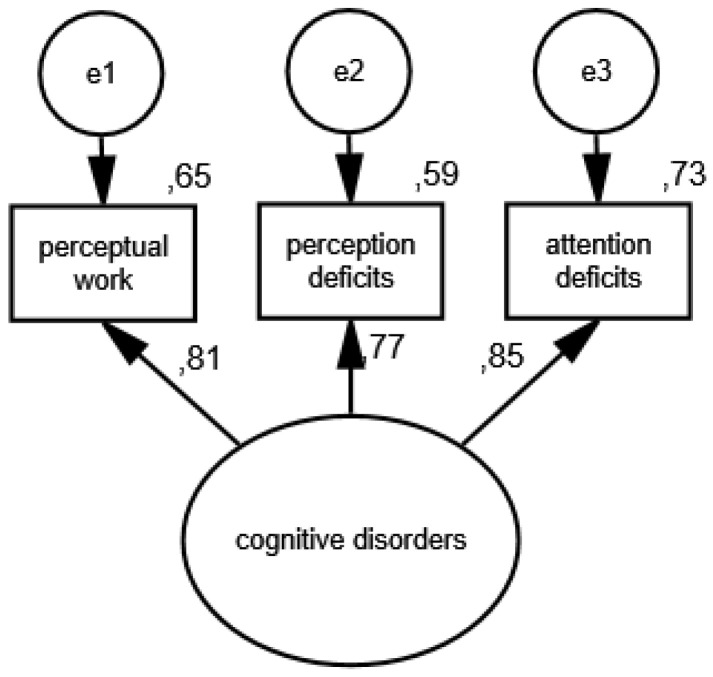
Standardized effects of the measurement model of cognitive disorders in the group of women.

**Figure 6:**
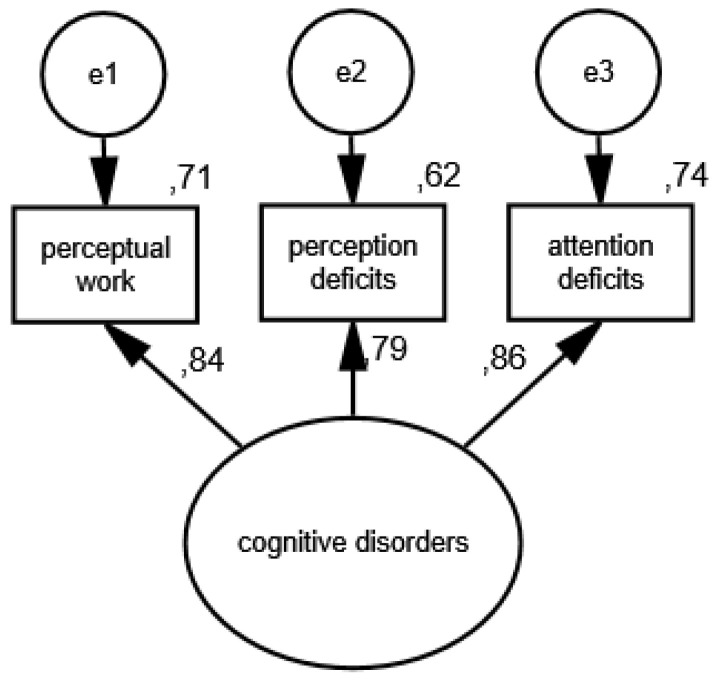
Standardized effects of the measurement model of cognitive disorders in the group of men.

In the third measurement model, the presence of cognitive disorders is an unobservable variable, and its partial indicators are perceptual work, perception deficits and attention deficits. The results of the verification of the constructed model for measuring cognitive disorders in the group of women are shown in Figure 5 and Figure 6 illustrates the measurement model of cognitive disorders in the group of men.

As a result of the estimation, the following measures were obtained: CMIN/DF = 1.75; GFI = 0.99; AGFI = 0.99; NFI = 0.99; RFI = 0.95; IFI = 0.99; TLI = 0.99; CFI = 0.99; RMSEA = 0.001(0.001; 0.05); PCLOSE = 0.92; *N* HOELTER(1317; 1596), which confirm that the model reflects the nature of the relationships between the analyzed variablesvery well. The standardized values of factor loadings and the percentages of variances explained in each of the analyzed groups show that the constructed model of cognitive disorders measurement is acceptable.

**Figure 7:**
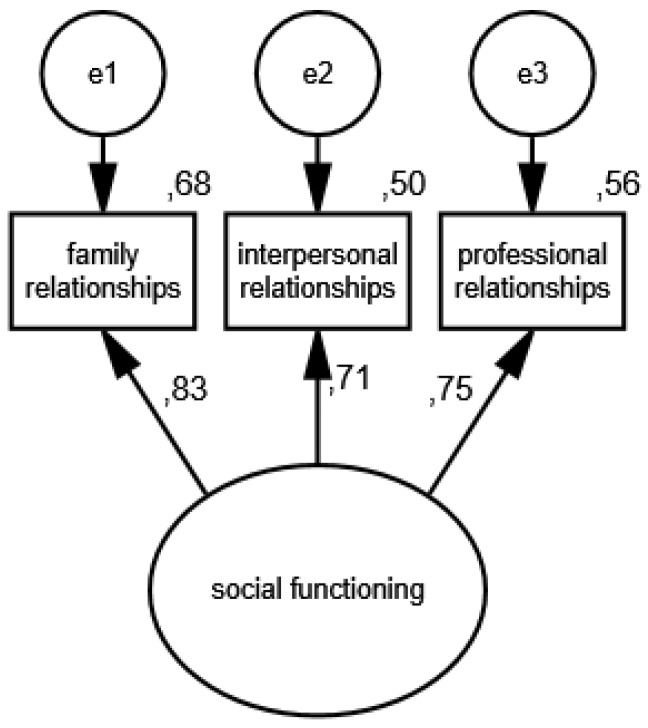
Standardized effects of the measurement model of social functioning in the group of women.

**Figure 8:**
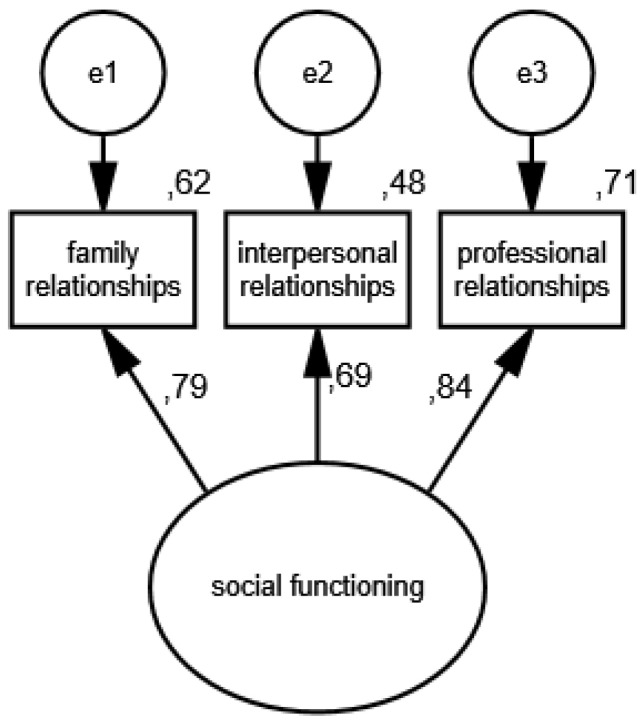
Standardized effects of the measurement model of social functioning in the group of men.

In the fourth measurement model, social functioning is an unobservable variable, and its partial indicators are support received from family members, friends, colleagues and medical professionals. The results of the estimation of the social support functioning model in the group of women are presented in Figure 7 and Figure 8 illustrates the measurement model of social functioning in the group of men.

The following measures were obtained: CMIN/DF = 1.75; GFI = 0.99; AGFI = 0.99; NFI = 0.99; RFI = 0.95; IFI = 0.99; TLI = 0.99; CFI = 0.99; RMSEA = 0.001(0.001; 0.05); PCLOSE = 0.92; *N* HOELTER(1335; 1428), which confirm its compliance with the data. The standardized values of factor loadings and the percentages of variances explained in the groups analyzed indicate that the constructed model of social functioning measurement is acceptable.

### 3.3. Structural Model Estimation

**Figure 9:**
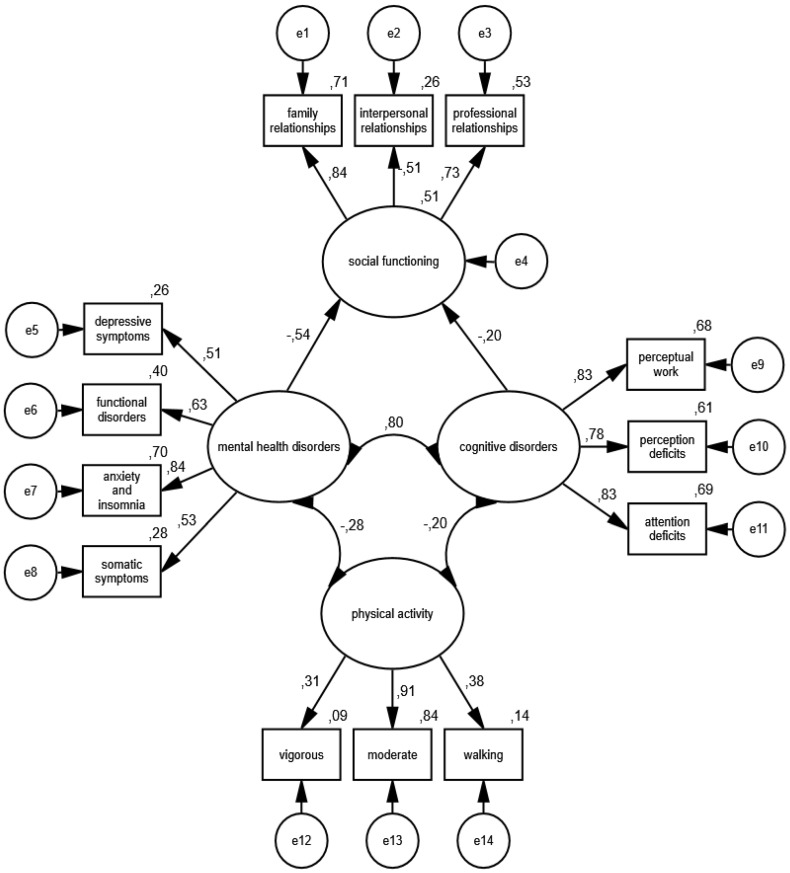
Standardized effects of the structural model of the relationship between physical activity and psychosocial functioning in a group of women.

**Figure 10:**
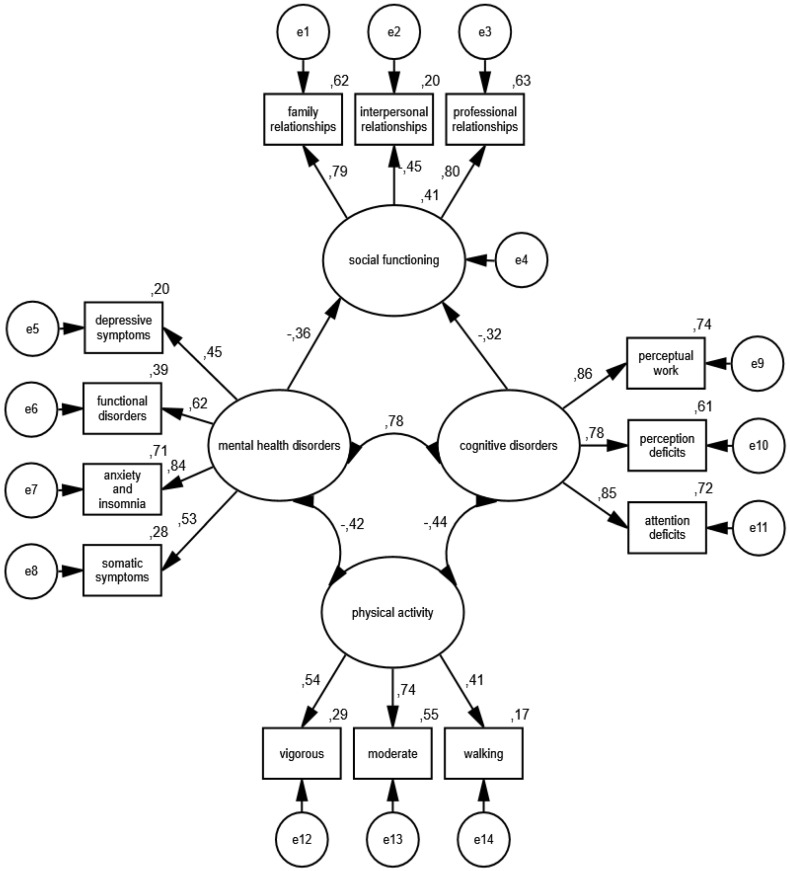
Standardized effects of the structural model of the relationship between physical activity and psychosocial functioning in a group of men.

The estimation and acceptance of the constructed measurement models allows for the estimation of the full interactive structural model, which was developed on the basis of the theoretical frameworks. As in the case of measurement models, its specification was carried out in the group of women and men. Figure 9 shows the construction and detailed standardized parameters of the interactive model of the relationship between physical activity, mental health, cognitive disorders and social functioning in the group of women, and Figure 10 depicts the results obtained in the group of men.

The accuracy of the interactive structural model was positively verified based on the following measures of goodness of fit: CMIN/DF = 1.38; GFI = 0.99; AGFI = 0.97; NFI = 0.98; RFI = 0.96; IFI = 0.99; TLI = 0.99; CFI = 0.99; RMSEA = 0.019 (0.008; 0.28); PCLOSE = 1.00; *N* HOELTER(1008; 1120).

In the group of women, the constructed model explained 51.0% of social functioning, which reflects the quality of tolerable family, interpersonal and professional relationships, and in the population of men, 41.0% of the variance of the analyzed variable was explained. In the group of women, there was a strong, directly proportional correlation between mental health disorders and cognitive disorders (*p* = 0.001), as well as a weak negative relationship of physical activity with mental health disorders (*p* = 0.001) and with cognitive disorders (*p* = 0.001). There was also a moderate, inversely proportional relationship between mental health disorders (*p* = 0.001) and social functioning, as well as a weak relationship between cognitive impairment and social functioning (*p* = 0.002).

In the group of men, there was a strong, directly proportional correlation between mental health disorders and cognitive disorders (*p* = 0.001), as well as a moderate negative relationship of physical activity with mental health disorders (*p* = 0.001) and with cognitive disorders (*p* = 0.001). There was also a moderate, inversely proportional relationship between mental health disorders (*p* = 0.001), cognitive disorders (*p* = 0.001) and social functioning.

The model shows that the consequences of the COVID-19 epidemic in Eastern Poland expressed in the form of mental health disorders and symptoms typical of cognitive disorders were moderated by physical activity from various sources. The more often the respondents engaged in physical activity by going for walks and training moderately and vigorously, the lower the risk was of developing mental disorders, manifested in the form of somatic symptoms, anxiety and insomnia, functional disorders and the symptoms of depression, as well as the occurrence of cognitive disorders, such as perceptual work disorders, perception deficits and attention deficits. Moreover, physical activity weakened the relationship between mental health disorders and disorders of cognitive processes and social functioning in family, interpersonal and professional relationships of the studied groups.

It should also be added that the comparisons of the values of parameters within and between groups showed that the relationship between mental health disorders and cognitive disorders was comparable in each group and stronger than the correlations between physical activity and mental health disorders and cognitive disorders. Moreover, in the case of women, the leading factor associated with the quality of social interactions was the state of mental health, which was less dependent on the level of physical activity than on the functioning of cognitive processes moderated by physical effort, while the quality of male social interactions depended on the relationship between the state of mental health and the cognitive functioning that is impaired by physical activity. In the population of women, compared to men, physical activity was less important for mental health and the functioning of cognitive processes, and the occurrence of symptoms typical of mental health disorders was more strongly associated with the quality of social interactions of women than men. It has also been shown that disorders of cognitive processes correlate more weakly with the social functioning of women than men.

## 4. Discussion

The outbreak of the COVID-19 pandemic caused severe and long-lasting stress in many people from different countries of the world, the relatively common consequences of which were mental health problems associated with deterioration of cognitive and social functioning [6,53,54]. Despite many published empirical studies devoted to the discussed issue, the role of physical activity in the prevention of psychosocial functioning of the inhabitants of Eastern Poland has not been analyzed so far [53,55,56].

Therefore, research was carried out to assess the level of physical activity, mental health, cognitive processes and social functioning, as well as the interactions and relationships between them, in the population of Eastern Poland, depending on gender.

With regard to the above goals, two hypotheses were formulated. The first of them assumed that during the COVID-19 pandemic, in the inhabitants of Eastern Poland, the level of physical activity and the severity of symptoms characteristic of mental health disorders, cognitive disorders and the quality of social functioning differed depending on gender.

The conducted analysis allowed us to positively verify the adopted assumption in terms of statistically significant gender differences regarding levels and severity of weekly activity, as well as the severity of vigorous activity, moderate activity, walking, mental disorders, somatic symptoms, functional disorders, depressive symptoms, cognitive disorders, perceptual work, attention deficits, family relationships and professional relationships. It has been shown that women were less likely than men to show a high level of physical activity, but more often their weekly activity ranked at sufficient and insufficient levels. It was also noted that surveyed men showed higher than average physical activity, walking, moderate physical activity and vigorous physical activity than women, and they functioned more efficiently in family relationships, but had more difficulty finding their way in professional interactions. Moreover, men showed a lower severity of mental disorders, somatic symptoms, functional disorders, depressive symptoms, cognitive disorders, perceptual work disorders and attention deficits as compared to women.

The men had a moderate state of mental health and cognitive processes, while women’s scores in mental disorders and perceptual work disorders were approaching the edge of the elevated area. The average weekly physical activity of men was high, and that of women was at a sufficient level, with the activity of more than three-quarters of the men surveyed being high, at nearly one-fifth sufficient, and 5.8% insufficient. In contrast, the women’s group reported markedly different results. Almost three-fourths of the female respondents showed a sufficient level of activity, while high and low activity levels were reported by 15.0% and 13.7% of the respondents, respectively.

Comparing the data obtained with the results of surveys conducted in other regions of Poland and the world, one can see significant discrepancies. The research already mentioned in the theoretical introduction proves that both men and women from the northeastern part of Poland manifest an insufficient and significantly understated level of physical activity in comparison to residents of other regions of this country and European countries. At the same time, men engaged in moderate physical activity and rode a bicycle more frequently than women [33].

Another empirical study also observed that women reported lower moderate physical activity than men, but were more likely to engage in light physical activity. It is worth noting, however, that because the study was conducted in a population of patients with a diagnosis of peripheral artery disease, vigorous activity was not analyzed in the study [57].

In contrast, a Canadian study, similar to our own, reported that women were less physically active than men during the COVID-19 pandemic, a result that was statistically significant [58]. Analogous observations have also come from the general population of Beirut [59]. In addition, women reported more barriers to engaging in systematic physical activity and reported significantly weaker mental fitness than the opposite sex [58,59]. A key observation appears to be that, among women, the most severe adverse mental health symptoms occurred in subjects whose physical activity was very low [58].

Thus, both the results of our own study and the conclusions of the work of other researchers suggest that, especially in the group of women, in the event of the occurrence or intensification of the impact of existing stressors, there may be a risk of developing undesirable symptoms in the state of mental health and cognitive processes [60]. This observation seems to be particularly important in the situation of a prolonged health crisis, because the effectiveness of coping, under the influence of chronic distress, may show a downward trend, and this directly implies an increased risk of undesirable symptoms in the field of mental health [61].

This effect was observed by Di Maio’s team. On the basis of the conducted moderation analysis, he noted that the belief in the possessed remedial abilities was directly related to physical activity in a situation where the encountered challenges were relatively low. On the other hand, when these challenges were high, the relationship between physical effort and faith in one’s abilities did not reach the level of statistical significance. The observed dependencies confirm the need to monitor not only direct but also long-term consequences in terms of the mental health of people affected by the COVID-19 pandemic [62].

The need to conduct this type of longitudinal research is also indicated by other researchers who, while forecasting the upcoming increase in the number of patients with depressive and anxiety disorders resulting from the current health crisis, as well as from the current problems of the mental health care system, point to the important role of physical activity in preventive interventions [7,63]. Systematic endurance training, by activating the HPA axis, stimulates the release of dopamine, which is responsible for self-regulation and mood [64,65]. The results of the meta-analysis, which included the data of 40,550 adults, show that the respondents who undertook systematic physical activity, compared to women and men with moderate and low activity, were less likely to develop mental disorders. The highest risk group was those who led a sedentary lifestyle [66], while the level of physical activity, physical fitness, mental health, cognitive processes and the quality of functioning in social interactions were higher in men than in women [67,68,69,70].

In this context, it should be noted, however, that the relationships between the aforementioned variables in the interactive and multidimensional approach have not been analyzed so far [71,72]. Taking into account the above premises, the second hypothesis assumed that during the COVID-19 pandemic in the inhabitants of Eastern Poland, depending on gender, the following are different: the importance of interactions between physical activity, mental health and cognitive disorders for social functioning.

The constructed structural model of the relationship between physical activity and selected aspects of psychosocial functioning allowed for a positive verification of the assumption. As a result of its estimation, in the group of women and men, the existence of interactions between physical activity, disorders of mental health and cognitive processes was confirmed, and their association with the social functioning of the analyzed groups was verified. Systematic physical activity, including intense and moderate endurance training and walking, was negatively associated with symptoms characteristic of mental health disorders, which include symptoms of depression, functional disorders, anxiety and insomnia and somatization, as well as cognitive disorders manifesting as weakened speed of perception and deficits in perceptiveness and attention to social functioning of the respondents.

In the case of women, the leading factor related to the quality of social interactions was the state of mental health, which was less dependent on the level of physical activity than on the functioning of cognitive processes, while the quality of men’s social interactions was most dependent on the relationship between the state of mental health and the functioning of cognitive processes. Moreover, in the group of women, compared to men, physical activity was more weakly linked to the state of mental health and the functioning of cognitive processes, and the occurrence of symptoms typical of mental health disorders had correlated more strongly with the quality of women’s social interactions than men’s. In turn, cognitive disorders were more weakly linked to the social functioning of women than men.

Relating the observed regularities to the conclusions of the empirical works of other researchers, it can be seen that they are to some extent similar [67,73]. In a sample of 5856 adults aged 26–70 years, it was noted that in the male population, physical activity was more negatively associated with problems in the sphere of mental functioning than in the group of women [67].

Another empirical study positively verified and moderated the role of gender in relation to physical activity, health-related quality of life and perceived social support. It was shown that people with the lowest assessments of mental health status had a 1.8 times lower probability of obtaining social support than respondents who showed high and average assessments of mental condition, while the participants with the lowest results in terms of physical health were twice as likely to be deprived of the help of their relatives than women and men who perceived their somatic health as very good or average. Moreover, men with low physical activity compared to women more often reported poor mental and somatic health and a lower overall quality of life than women. It should be added, however, that the discussed model did not take into account the symptoms characteristic of cognitive disorders [73].

Summing up the discussion, it is worth emphasizing that the results of our own research seem promising in the context of preventing the psychosocial functioning of adult inhabitants of Eastern Poland who have been affected by the COVID-19 epidemic. Nevertheless, due to the existing limitations, the observed effects require further empirical verification.

The aforementioned limitations include the fact that, in order to minimize the risk of SARS-CoV-2 infection and taking into account the epidemic restrictions in the country, the study was conducted online, which might not have been fully comfortable for the participants. In addition, the study sample consisted of volunteers, who had access and the ability to use a computer, which, despite a relatively large number of observations and controlling for a number of side variables, could have affected the results. Consequently, the data may not fully reflect the psychosocial situation in the Eastern region of Poland. In addition, the study population was made up exclusively of people who were employed and those in marriages and partnerships. This suggests, therefore, that the research results obtained should be generalized, with some caution, only to the population of healthy, economically active residents of Eastern Poland with life partners.

It should also be added that there were no people infected with the SARS-CoV-2 coronavirus and COVID-19 patients among the participants, so in the subsequent studies, it would be worth verifying whether this factor differentiates mental health disorders, cognitive disorders and social functioning. Another area requiring exploration is the determination of the level of physical activity and these parameters and the relationships between them in the population of medical staff, especially those who specialize in COVID-19 treatment. Due to the specificity of their work, the indicators of physical activity, mental health and cognitive problems as well as social functioning may be higher in this group than in the general population. It is also important to mention that the region of Eastern Poland is characterized by a relatively low transmission of the SARS-CoV-2 coronavirus; therefore, the psychosocial consequences of the epidemic may be lower in the inhabitants of this part of the country than in those affected by a higher epidemic risk.

Another aspect that should be mentioned in the context of discussing the limitations of the presented results is that due to the cross-sectional nature of the research and the lack of data from before the outbreak of the pandemic, the conducted analysis showed only the current intensity of individual parameters and the strength of the relationships between them; however, the dynamics of changes under the influence of SARS-CoV-2 transmission were not estimated. For this reason, it is not possible to precisely determine to what extent the current pandemic situation has influenced the change in the intensity of the analyzed variables and the strength of their relationships.

It is also worth mentioning that the measurement of physical activity was carried out using the short form of the questionnaire (*IPAQ-SF*), which may have made it slightly less reliable than the full version (*IPAQ*) [25,50]. However, the decision to choose the *IPAQ-SF* was based on the suggestions of the authors of the Polish adaptation of the test, who, based on the conclusions of a number of empirical studies, recommend the use of the abbreviated Polish version of the tool for research conducted both within one country and conducted internationally. They point out that the full version of the *IPAQ*—due to its detail—requires much greater involvement of the subjects, and this in turn directly implies impatience, a tendency to give random answers, skip questions or drop out of the survey [50]. Researchers from other regions of the world have also encountered similar problems with the full version of the test [25]. Given that in our study, in addition to the test used to measure physical activity, four other questionnaires were used, the aforementioned risks were reduced by using the *IPAQ-SF* [25,26,27,28,29,30,31,32,33,34,35,36,37,38,39,40,41,42,43,44,45,46,47,48,49,50].

Nevertheless, the presented study is the first and largest project focused on the analysis of the psychosocial functioning of the inhabitants of Eastern Poland during the COVID-19 epidemic, in which diagnostic tools with high psychometric properties and advanced statistical methods were used. This allowed for the visualization of the structure of mutual and multifaceted relationships between particular variables in the group of women and men.

Moreover, the discoveries are of great cognitive and practical importance. Determining the current level of psychosocial functioning of the inhabitants of Eastern Poland during the pandemic, in terms of the level of physical activity, mental health, cognitive processes and the quality of social functioning, as well as verifying the occurrence and significance of interactions and relationships depending on gender, not only enriches knowledge about the mechanisms shaping the responses of adults to the current health crisis, but it is above all the basic element that allows for taking effective preventive actions. Confirmation that various forms of physical activity are a factor protecting against negative effects on mental health, cognitive processes and social functioning may constitute the basis for developing gender-individualized, interdisciplinary psychopreventive interventions aimed at strengthening the intrapsychic and interpersonal spheres of women and men at risk of the negative consequences of SARS-CoV-2 transmission.

## 5. Conclusions

The conducted analyses allowed us to determine the current level of psychosocial functioning of the inhabitants of Eastern Poland, as well as the interactions and relationships between physical activity, disorders of mental health and cognitive processes and social functioning during the COVID-19 epidemic, which in turn allowed for the formulation of the following implicative conclusions:

- Taking into account that the population in question included people at risk of developing problems in psychosocial functioning, it would be important to support the analyzed population with an appropriate preventive program;
- Women, as compared to men, show a lower level of physical activity, including weekly activity, walking, moderate activity and vigorous activity, which may be a result of different uses of time in the course of a day, and for women, is associated with higher recorded intensity of symptoms common to mental health disorders, especially somatic symptoms, functional disorders and depressive symptoms, as well as cognitive disorders—in particular those related to perceptual work and attention deficits, and also lower quality of functioning in family relationships, although higher coping skills in professional interactions are also seen; as a result, the developed program in the case of women should include interventions to support effective time management, and it is necessary that it be adjusted to the specifics of individual differences noted between women and men;
- Whereas physical activity, interacting with mental health disorders and cognitive disorders, weakens their coexistence and the relationship with social functioning, it is important to indicate that systematic exercise training adapted to the state of health and abilities of the organism may be an important element of psychoprophylaxis programs;
- Taking into account that in the male population, the negative associations of physical activity with symptoms typical of mental health and cognitive disorders are stronger than in the female population, while women’s social functioning depends more on their mental health than on their cognitive deficits, and the quality of men’s family relationships and interpersonal and professional relationships is similarly linked to mental health and cognitive disorders, the developed preventive interventions should be implemented early and tailored to the individual needs of the participants, which may translate into an amplification of their benefits.

In conclusion, it appears to be important to develop a systematic and interdisciplinary psychoprophylactic physical training program individualized based on gender and health condition. Designing this type of intervention would provide an opportunity to improve the intrapsychic, cognitive and social functioning of Eastern Poland’s population during the COVID-19 epidemic.

## Figures and Tables

**Table 1:** Sociodemographic characteristics of the surveyed women and men.

Variables		Group				Group Comparison	
		Women		Men			
		*M*	*SD*	*M*	*SD*	*t*	*p*
Age		46.79	11.92	46.35	11.48	0.40	0.691
Number of children		2.39	1.02	2.22	1.12	1.71	0.09
		*n*	*%*	*n*	*%*	*x* ^2^	*p*
Place of residence	City	113	50.0	113	50.0	0.00	1.00
	Village	113	50.0	113	50.0		
Education	Primary	27	11.9	37	16.4	5.29	0.152
	Vocational	81	35.9	72	31.9		
	Secondary	78	34.5	64	28.3		
	Higher	40	17.7	53	23.4		
Marital status	Married	113	50.0	113	50.0	0.00	1.00
	Informal relationship	113	50.0	113	50.0		
Professional activity	Employment contract	113	50.0	113	50.0	0.00	1.00
	Contract of mandate	113	50.0	113	50.0		

Markings: *M*—mean, *SD*—standard deviation, *t*—value of Student’s *t*-test for independent data, *p*—significance level.

**Table 2:** Comparison of physical activity levels of women and men.

Variable		Group				Group Comparison		
		Women		Men				
		*n*	*%*	*n*	*%*	*x* ^2^	*p*	*V*
	High	34	15.0	174	76.9	156.53	0.001	0.59
Physical activity *	Sufficient	161	71.3	39	17.3			
	Insufficient	31	13.7	13	5.8			

Markings: *n*—count, *%*—percent, *x*^2^—c^2^ test value, *p*—significance level, *V*—effect size expressed by Crammer’s *V* coefficient, *—levels of weekly physical activity were distinguished based on results expressed in MET units.

**Table 3:** Comparison of physical activity, mental health, cognitive processes and social functioning of women and men.

Variables		Group				Group Comparison		
		Women		Men				
		*M*	*SD*	*M*	*SD*	*t*	*p*	*d*
	Weekly activity	840.31	1013.04	1593.92	1244.42	5.97	0.001	0.56
Physical activity *	Walking	396.59	642.54	583.49	821.69	2.30	0.022	0.22
	Moderate activity	159.33	262.53	561.97	541.13	8.73	0.001	0.82
	Vigorous activity	285.39	381.90	448.45	591.34	2.98	0.003	0.28
	Sitting, lying down	228.88	101.39	209.01	87.94	1.85	0.66	-
	Overall Score	10.70	6.01	7.47	7.12	5.17	0.001	0.49
Mental health disorders **	Somatic symptoms	3.16	1.95	1.88	2.41	6.20	0.001	0.58
	Anxiety and insomnia	2.79	2.63	2.45	2.79	1.73	0.084	-
	Functional disorders	2.26	2.55	1.39	2.50	3.68	0.001	0.35
	Depressive symptoms	2.48	1.61	1.86	1.96	3.65	0.001	0.34
	Cognitive functioning	6.28	0.79	5.78	1.10	5.67	0.001	0.53
Cognitive disorders ***	Perceptual work	6.70	1.20	5.90	1.49	6.28	0.001	0.59
	Perception deficits	6.23	1.33	6.38	1.54	1.04	0.298	-
	Attention deficits	5.92	1.35	5.05	1.61	6.15	0.001	0.58
	Overall Score	4.93	1.71	4.70	1.41	1.24	0.215	-
Social functioning ***	Family relationships	4.77	1.85	5.70	1.73	4.98	0.001	0.47
	Interpersonal relationships	4.74	1.79	4.87	1.95	0.65	0.514	-
	Professional relationships	4.32	2.64	3.04	2.29	5.56	0.001	0.52

Markings: *M*—mean, *SD*—standard deviation, *t*—value of Student’s *t*-test for independent data, *p*—significance level, *d*—measure of Cohen’s *d* effect size, *—results expressed in MET units, **—raw results, ***—results expressed in sten.

## Data Availability

Data supporting the results of this study are available from the corresponding author (AM) upon request.
